# Practicality of 3D Printed Personalized Medicines in Therapeutics

**DOI:** 10.3389/fphar.2021.646836

**Published:** 2021-04-12

**Authors:** Hilda Amekyeh, Faris Tarlochan, Nashiru Billa

**Affiliations:** ^1^ Department of Pharmaceutics, School of Pharmacy, University of Health and Allied Sciences, Ho, Ghana; ^2^ College of Engineering, Qatar University, Doha, Qatar; ^3^ College of Pharmacy, QU Health, Qatar University, Doha, Qatar

**Keywords:** 3D printing, personalized medicine, pharmacogenomics, quality assurance, dosage forms, practicality, tablets, formulation

## Abstract

Technological advances in science over the past century have paved the way for remedial treatment outcomes in various diseases. Pharmacogenomic predispositions, the emergence of multidrug resistance, medication and formulation errors contribute significantly to patient mortality. The concept of “personalized” or “precision” medicines provides a window to addressing these issues and hence reducing mortality. The emergence of three-dimensional printing of medicines over the past decades has generated interests in therapeutics and dispensing, whereby the provisions of personalized medicines can be built within the framework of producing medicines at dispensaries or pharmacies. This plan is a good replacement of the fit-for-all modality in conventional therapeutics, where clinicians are constrained to prescribe pre-formulated dose units available on the market. However, three-dimension printing of personalized medicines faces several hurdles, but these are not insurmountable. In this review, we explore the relevance of personalized medicines in therapeutics and how three-dimensional printing makes a good fit in current gaps within conventional therapeutics in order to secure an effective implementation of personalized medicines. We also explore the deployment of three-dimensional printing of personalized medicines based on practical, legal and regulatory provisions.

## Introduction

Three-dimensional printing (3DP) has been in existence for decades but has regained attention more recently due to the huge potential it holds to addressing several of the constraints associated with the current modality in therapeutic interventions. For example, conventionally manufactured dosage forms like tablets and capsules are regimented with a fit-for-all provision. Clinicians have limited options when gauging the required dose (based on the severity of the disease) from unit dose medicines like tablets or capsules. This situation is less acute with continuous dosage forms like syrups and suspensions. Limitations in calibrated dosing may poses constraints when prescribing/dispensing, which may lead to sub-therapeutic levels or potentially toxic blood levels in patients. When we take into account the fact that patients express various levels of metabolizing enzymes such as CYP 450 and hence respond differently to the same treatment scheme (Vogenberg et al., 2010; Abul-Husn et al., 2014), the preceding scenario is all the more crucial. As a result, pharmacogenomics, which deals with the interplay between genetic variations in individuals and their responses to medication, is recognized as key to ensuring safety in therapeutics. Several researchers have devoted their attention to unlocking many genetic codes that play a crucial role in drug metabolism.

The above interest has led to the evolution of the concept of personalized medicine (PM), where therapy can be tailored to individual responses, with the highest possible safety margins. Furthermore, PM is cost-effective since patients are not exposed to unnecessarily high doses (Vogenberg et al., 2010). PM, also referred to as precision medicine, guides clinicians in selecting appropriate therapeutic agents in the right dose for specific patients in specific disease states. This interplay also allows effective monitoring of patient condition. However, in terms of practicality of PM, genetic profiling should be done *en masse* and clinicians must have ready access to patient and health records. Furthermore, current regulatory barriers on use of patient personal information in medical diagnosis should be permissive. Current trends indicate that PM would revolutionize conventional treatment interventions (Vogenberg et al., 2010; Salari and Larijani, 2017), as we realize that therapy is evolving into trends that enable best clinical outcomes. This is affirmed because our knowledge of the human genome and metabolic pathways has increased significantly over the past decade. Regulatory authorities such as the United States Food and Drug Administration (FDA) have recognized this paradigm shift in therapeutics and have made inroads to accommodate innovations in this regard (Basit, 2020). In fact, the FDA has approved some personalized medicines, Spritam^®^ for example, manufactured by Aprecia Pharmaceuticals Company, with the possibility of several more in the pipeline.

In tandem to the growing need for versatility in the application of PM is the growth and advancement of delivery devices that complement the deployment of PM. Ideally, these devices and their construction should be amenable to the demands of PM. Over the past decade, 3DP has emerged as one of the more realistic approaches to addressing the requirements of PM and has won the interest of researchers and clinicians alike as a contender in the PM frontier.

One major benefit of 3DP in pharmaceutical applications is the potential of fabricating drug products that have complex geometries. Additionally, customization of dosage forms can be achieved with 3DP, with a typical example being printing of implants to fit the contour of the implantation site on a patient’s body (Wu et al., 2009; Lim et al., 2018). The technique can also be used to achieve on-demand production of dosage forms at a healthcare or production facility. Furthermore, it may be cost-effective to use the technology to produce small quantities of drug products (Lim et al., 2018).

There are several modalities of 3DP, but in the context of PM, we are concerned with the printing of drug-loaded devices tailored to the needs of patients. A review of relevant 3DP techniques is presented in *Techniques With Relevance to Three-Dimensional Printing*. This review aims to shed light on the practicality of 3DP within the context of PM for in-patient and out-patient settings and offer practical solutions where gaps are apparent.

## Conventional Therapeutics

Medicines have historically been manufactured with strong considerations on pharmaceutical attributes of the finished product as well as amenability for automatized production. These considerations suit mass production lines within the pharmaceutical industry, particularly for solid dosage forms like tablets and capsules. Solid dosage form manufacturing is tuned for mass production of single unit doses. Indeed, equipment used in production of tablets and capsules can be calibrated to produce hundreds of specific dose units per minute with extreme precision. These unit doses are largely dictated by proprietary provisions with little, if any, consideration to clinical manifestations (Yuen et al., 2001). The manufacturing of tablets typically follows a sequence of processes including mixing, granulation, drying, and finally compression. Capsules are made by filling empty hard gelatin capsules with granules to the required dose of the active pharmaceutical ingredient (API). The performance of the API is ascertained *a priori* via bioequivalence comparisons with a propriety formulation. Whilst such formulations are considered “bioequivalent”, clinically relevant data are mainly attributable to the proprietary formulation. Considerations to substitute a generic formulation with the proprietary formulation are solely based on pharmacokinetic data. As mentioned in the Introduction, such fit-for-all production framework does not take into account genetic variations amongst patients, whereby a standard dose may be alright in some individuals but toxic in others. Furthermore, epigenetic factors are not given due consideration during dosing from conventional therapeutics. The concept of PM is well primed to fill this gap as is discussed in the following section.

## Personalized Medicine

PM refers to customization of medical treatment to the needs, characteristics, and preferences of individual patients (Redekop and Mladsi, 2013). It is a medical practice in which the “one-size-fits-all” approach is replaced with the “right drug” in its “right quantity” for the “right patient” at the “right time” (Annas, 2014). The right drug for one patient may be the one with the least side effects. PM is based on the fact that each individual’s genome specifies their response, including adverse effects and allergic reactions, to specific drugs, diets, and lifestyle activities, which are all crucial in disease management. As a result, PM is a medical approach in which the diagnosis, prevention, and treatment of health conditions are based on inter-individual genetic differences (Salari and Larijani, 2017).

Typically, homogenous liquid dosage forms are suitable for personalized dosing based on appropriate volume-dose calculations (Brown et al., 2004). The volumes of liquid medicines can be accurately measured using accurate and affordable dosing devices that usually accompany the medicines. However, these dosing aids have been associated with several potential sources of inaccuracies, such as devices with wrong volumes, counting errors for drops, shape effects of spoons, and confusing graduations on syringes and measuring cups (Grießmann et al., 2007; Yin et al., 2010; Walsh et al., 2011). Furthermore, manual dexterity and cognition are needed for patients or their caregivers to accurately dose liquid medicines (Peek et al., 2002).

In contrast to liquid dosage forms, solid dosage forms are typically manufactured in predefined doses or strengths based on the findings from clinical trials for therapeutic effects in majority of the population (Herxheimer, 1991; Cohen, 2001). However, following approval by regulatory agencies for commercialization, these products are used among larger and more diverse populations. The inflexibility of these fixed-dose products starts manifesting once they are available on the market. For instance, fluoxetine (Prozac^®^) was initially manufactured as a 20 mg tablet, as that was found to be suitable for 64% of the target population. However, at a lower dose of 5 mg, it was found that 54% of the population still had a beneficial effect with fewer adverse effects (Cohen, 1999). The antihypertensive medicine atenolol was initially introduced as 100 mg tablets. However, years later, 50 and 25 mg tablets were introduced as they were more suitable for elderly patients (Herxheimer, 1991). Interestingly, there are other instances in which required strengths/doses of solid dosage forms for specific patients may not be available (Pies, 1995), which can negatively impact the attempt to effectively individualize pharmacotherapy for better patient care.

It has been indicated that the prevalence of adverse effects due to untailored therapy is 75–85% (Cohen, 1999). This is because patients’ responses to drug doses vary widely; as a result, some populations may experience the desired therapeutic outcome, whereas others may experience adverse effects or have inadequate plasma drug levels for therapeutic effects (Cohen, 2002). Moreover, responses to mass-manufactured discrete drug doses can vary 10–30 fold or more among the majority of patients (Cohen, 1999; Ma and Lu, 2011). Additionally, polypharmacy and co-morbidities usually complicate treatment, particularly in the elderly (Florence and Lee, 2011), which can further make the use of separate fixed-dose medications cumbersome, untailored, and ineffective. These can result in poor patient compliance and increase the risk of medication errors. Therefore, in such an instance, multiple medications needed by a particular patient can be combined into a single tablet for personalized dosing (Lim et al., 2018). PM has a high potential in the tailoring of therapy to achieve the highest safety margin and the best response to pharmacotherapy to ensure better patient care. This is because optimal risk assessments can be performed and diseases can be diagnosed earlier in PM practice. PM also holds promise for lowering healthcare costs while improving healthcare, as unnecessary costs associated with the trial-and-error approach in medical processes could be avoided (Vogenberg et al., 2010; Lim et al., 2018).

In the pharmaceutical and biomedical industries, device and drug manufacturers can use the principle of PM to develop medications for specific groups of patients who do not respond to drug agents as majority of the population does, and for whom conventional approaches to managing their health conditions have failed. However, successful practice of PM requires changes in practice patterns and management strategies, as well as new value assessments for products to be used. Notwithstanding, all the relevant stakeholders will need to address barriers to PM implementation to achieve individualized diagnoses, prognoses, and treatments (Vogenberg et al., 2010).

### Prospects of Pharmacogenomics and Dosing Accuracy

PM relies significantly on pharmacogenomics, which is an advancement in medical science that seeks to focus on diseases at the molecular level to understand the underpinnings of drug response. This is because genome information is translated into public health and clinical practices in PM. Pharmacogenomics involves identification of genetic predispositions, understanding their implications, and using them to achieve better preventive measures, better diagnostic assessments, and better therapeutic interventions. These can lead to improved drug efficacy as well as fewer and/or ameliorated adverse effects to medications (Schleidgen and Marckmann, 2013). Overall, a hopeful transition from current medical practices to PM has the prospect of improving clinical practice (Abettan, 2016) and human health (Green et al., 2011).

Drugs generally undergo rigorous testing and approval processes; however, there is often no way to predict how each individual will react to a specific agent. Consequently, several patients die from adverse reactions to medications, whereas thousands are hospitalized (Vogenberg et al., 2010). A medication may be safe for most people but cause toxic reactions in others because of interindividual variations in genes. Pharmacogenomics can be used to predict those who are likely to have a bad reaction to a drug before they consume it, and those who will be likely to respond to a medication successfully. It can therefore be used to improve drug discovery and development. Specifically, drugs can be designed to overcome resistance, have new targets, or minimize variations in their blood levels by optimizing their pharmacokinetics (Vogenberg et al., 2010; Ma and Lu, 2011).

Furthermore, pharmacogenomics could potentially help pharmaceutical companies to recuperate monies spent on orphan drugs, which are products that have been abandoned because of unprofitable cost projections or the side effects they caused in clinical trials. Pharmaceutical companies may be able to salvage these drugs by labeling and marketing them directly to specific populations that may benefit from them based on PM (Lee, 2005). During drug development, pharmacogenomic testing could be used in clinical trials to identify potential study participants with genotypes that are associated with an intended drug response. The costs and time involved in participant recruitment for phase II and III clinical trials could then decrease by using a study population comprised of individuals who have desired candidate genotypes (Lee, 2005). Moreover, pharmacogenomics may help pharmaceutical companies focus testing of their products, as persons with specific genetic variations that can result in adverse reactions or poor efficacy can be excluded from clinical trials, which is an important step to achieving PM goals. This can also help to speed up the clinical trial process for a target population (Vogenberg et al., 2010). Consequently, drug doses can be easily personalized for patients in these special populations.

Significant benefits of pharmacogenomics and PM include better medication selection, improvements in drug development, and safer dosing options. Currently, drug dosing follows the standard one-size-fits-all approach in most instances or is based on vital factors such as liver or kidney function, weight, and age. As useful as these parameters are, they may not be sufficient in achieving the best drug doses for individual patients. Pharmacogenomics can be used to predict the optimal dose of medication a person needs (Vogenberg et al., 2010).

PM is an extension of traditional approaches to understanding and treating diseases but with greater precision since treatment selection is guided by a profile of variations in a patient’s genes to ensure more successful outcomes. It is participatory, engaging patients in lifestyle choices and active health maintenance to compensate for genetic susceptibilities. In the future, PM will hopefully and simply be referred to as “medicine”, when the infrastructures needed are put in place, when diseases are classified and treated based on signs and symptoms as well as genetic and molecular profiles, when physicians make decisions on treatments based on their knowledge and a patient’s genomic information, when insurance companies pay for tests and treatments based on patients’ needs, and when regulators insist on the usage of “all” information available to the physician, including results of genetic tests, to ensure the safety and efficacy of an approved drug (Vogenberg et al., 2010).

Metabolomics, proteomics, and pharmacoepigenetics can be used together with pharmacogenomics to achieve individualized pharmacotherapy. Combining relevant principles in these areas can facilitate the study of genetic factors associated with multifactorial diseases and drug responses. Therefore, more clinical trials must be conducted to investigate the use and cost of genotyping and personalized medicines to obtain relevant data for clinical application (Ma and Lu, 2011). The economic implications of pharmacogenetics-based therapies are greatly influenced by the cost of genetic tests. It is estimated that these tests can cost between US$33 and US$710, with a wide variation in prices even for the same drug. However, average costs of testing are higher in the United States and Canada compared to other regions (Verbelen et al., 2017). However, the cost of panel-based genetic tests has been decreasing over time and with increasing availability. Therefore, it is becoming cost-effective to conduct these tests prior to diagnosis and treatment (Ormond and Cho, 2014; Verbelen et al., 2017). A common challenge when implementing pharmacogenetic testing is the need for rapid return of results. Fortunately, genotyping platforms that offer rapid sample-to-result assays are being developed (Abul-Husn et al., 2014).

Supporting evidence for the clinical application of pharmacogenetics can be obtained from randomized controlled trials (Abul-Husn et al., 2014). For instance, it has been revealed from retrospective studies that warfarin dosing based on a patient’s pharmacogenetic testing of *CYP2C9* and *VKORC1* is more accurate than fixed dosing or using dosing tables in warfarin labels (Finkelman et al., 2011). There are several other clinically relevant drugs such as antidepressants, benzodiazepines, antiretrovirals, anticancer drugs, proton pump inhibitors, mephenytoin, and clopidogrel, among others, which are considered for PM because certain enzymes and their variant alleles are involved in their metabolism (Abul-Husn et al., 2014). Specifically, reassessment of antiplatelet therapy with clopidogrel after genetic evaluation has resulted in successful implementation of CYP2C19*2 genetic testing for patients following percutaneous coronary intervention (Roberts et al., 2012).

Dosing accuracy in the drug delivery context refers to deviation of a predicted dose from the observed one. Personalized dosing as a result of PM can significantly improve dosing accuracy. Variations in responses to drugs are affected by differences in genetic and epigenetic factors, age, nutrition and health status, environmental influences, and concurrent therapy. Therefore, in order to achieve individualized drug therapy, which involves administering accurate drug doses to specific patients, with a reasonably predictive outcome, differences in drug response patterns among geographically and ethnically distinct populations must be considered (Vogenberg et al., 2010). The practice of PM can help to achieve a higher value in healthcare because PM can help to achieve more accurate dosing following a relatively inexpensive genetic test. It is estimated that about 1 billion USD of healthcare costs might be saved per year with PM while delivering quality care (Leavitt and Kucherlapati, 2008).

### Challenges Associated With the Practice of Personalized Medicine

Major concerns in PM implementation involve issues with the acceptance of the practice by various stakeholders in the healthcare system, including patients (Goetz and Schork, 2018). It may be challenging to educate and train healthcare professionals to practice PM. This is because, unlike in conventional practice, in PM, practitioners have the added responsibility of characterizing individual patients based on genomic, biochemical, and behavioral factors, among other dynamics, before selecting appropriate treatments for them (Goetz and Schork, 2018). Furthermore, the application of pharmacogenomics in PM requires large data collections before interindividual differences can be identified to guide treatment with specific interventions. This could raise privacy and legal issues about personal data due to concerns over such information being used for fraudulent purposes (Shen and Ma, 2017; Vayena and Blasimme, 2017; Mooney and Pejaver, 2018). Moreover, there are millions of genetic variations that must be identified before PM can be fully and effectively implemented. How an individual responds to a medication might not involve only one gene but the interaction of many genes (Vogenberg et al., 2010; Ginsburg and Willard, 2013). Therefore, searching through genetic profiles and maps, and understanding their implications prior to making a diagnosis or initiating a treatment may be expensive and time-consuming. It is however still anticipated that pharmacogenomics would be integrated into current medical practices to improve healthcare. Additionally, the accuracy and validity of genomic testing continues to improve; however, there are challenges with respect to interpretation and communication of test results (Vogenberg et al., 2010; Abul-Husn and Kenny, 2019).

Another challenge to effective PM practice is obtaining approval from regulatory agencies for the production and use of personalized dosage forms (Goetz and Schork, 2018). These concerns revolve around a need to prove that PM strategies are better than conventional medicine strategies, which is very crucial considering that tailored therapies may be more expensive. Additionally, more efficient strategies must be developed to meet the needs of specific patients as the practice progresses (Check Hayden, 2016; Hughes, 2018). Another challenge in PM realization for hospital management, healthcare providers, and health plan sponsors is implementation of health insurance schemes with respect to the regulatory and legal constructs of PM. This is because current structures and payment systems for healthcare, especially with respect to genetic tests and personalized treatments, must be redesigned to fit PM practice (Vogenberg et al., 2010).

## Therapeutic Potential of Three-Dimensional Printing Used in Conjunction With Personalized Medicine

3DP, also known as additive manufacturing, is a type of manufacturing technique in which an object is formed, usually based on digital models, by successive addition of layers of materials using a 3D printer (Prasad and Smyth, 2015). It can be used for various applications in healthcare as well as in the clothing, food, chemical, automobile, aerospace, and toy industries, among others.

In the pharmaceutical industry, 3DP can be used to produce personalized medicines and medical devices to achieve tailored treatment, which can help to improve patient compliance, adherence to therapy, and patient care in general (Norman et al., 2017). This can significantly help to achieve PM objectives because dosage form characteristics such as drug release profiles can be tailored to the therapeutic, diagnostic, and nutritional needs of individual patients (Pravin and Sudhir, 2018). Ideally, a dosage form that is fabricated to exhibit a pre-determined drug release profile will undergo optimal drug absorption and distribution and exhibit improved drug safety and efficacy (Moulton and Wallace, 2014).

The therapeutic potential of 3DP in combination with PM is remarkable because complicated structures can be fabricated using raw materials in various physical forms (Guo and Leu, 2013) to meet specific patient needs. Typically, this starts with the construction of a computer-aided design or model that specifies the physical characteristics of the final object, such as shape and size, to precision (Goyanes et al., 2015d).

Approval of the first 3D printed pharmaceutical product Spritam^®^, a powder-layered tablet produced by Aprecia Pharmaceuticals Company, in 2015 (United States Food and Drug Administration, 2015), as well as approval of OsteoFab Patient-Specific Facial Device (United States Food and Drug Administration, 2016) and OsteoFab Patient-Specific Cranial Device (United States Food and Drug Administration, 2013) produced by Oxford Performance Materials are clear indications that 3DP is a key technology for the manufacture of solid dosage forms and medical devices. More importantly, the technology has been used to fabricate various drug delivery devices aimed at individualized treatment of patients.

### Techniques With Relevance to Three-Dimensional Printing

#### Hot Melt Extrusion

HME is a common and widely used technique in 3DP. It works on the mechanism of automated extrusion of a melted substance through nozzles (Goyanes et al., 2015d). It usually requires supports such as scaffolds for the printing of drug to hold the extruded material in shape (Sadia et al., 2016). HME is a manufacturing process that includes several operations such as feeding, heating, mixing, and shaping in one continuous process (Feng and Zhang, 2017). The technology has received much attention in the medical and pharmaceutical industries for the production of pharmaceutical dosage forms and medical implants (Maniruzzaman et al., 2012).

In HME, materials are heated until they melt or soften (hot melt), after which pressure is applied to the molten material through an orifice/die to produce a material of uniform shape and density. The extrusion process can change the physical properties of a substance as it is forced through the orifice under controlled conditions (Patil et al., 2016). The main component of HME is the extrusion process. The basic parts of an extruder are the motor, extrusion barrel, rotating screws in the barrel, and a die or orifice at the end of the extruder. Process parameters that must be controlled in HME are screw speed (revolutions/minute), feed rate, temperature along the barrel and the die, and the vacuum level for devolatilization. The four different extrudate shapes that can be produced from the extrusion dies are strands, films, sheets, and granules (Patil et al., 2016).

It has been shown that HME can be used to improve the solubility and bioavailability of poorly soluble drugs (O’Connor and Lee, 2017; Repka et al., 2018). Moreover, the ability and efficiency of HME in producing solid dispersions has made it possible for the development of sustained, modified, and targeted drug delivery systems (Patil et al., 2016). There are drawbacks in the application of HME for the production of dosage forms. For example, the process requires high energy input, which may also cause thermal degradation to some APIs and excipients, which are usually polymers. The high shear forces may also cause physical degradation to the polymers. However, proper formulation and equipment design, as well as different engineering approaches, are being used to improve these shortcomings (Repka et al., 2008).

HME technology was originally used to manufacture plastic and rubber products (Zhang et al., 2017); however, it can be applied in the production of filaments required in 3DP of dosage. In HME, APIs are blended with a thermoplastic polymer, after which the mixture is softened/melted and extruded as filaments for use in 3DP (Tan et al., 2018).

#### Fused Deposition Modeling

FDM is an inexpensive 3DP manufacturing process that is an attractive alternative to the conventional methods of producing solid dosage forms. It is considered the lowest cost and easily accessible 3DP technology (Tan et al., 2018). The FDM process involves extrusion of a melted thermoplastic polymer filament by two rollers through a high temperature nozzle, followed by solidification of the melt on a build platform. The nozzle moves horizontally, whereas the build platform moves vertically downwards as the process continues. The tip of the nozzle used in FDM is typically in the range of 50–100 μm (Goyanes et al., 2014). After each layer is deposited, the build platform moves downwards for another layer to be deposited on the previous layer. Printing speed in FDM has to be optimized because it may significantly affect the thickness of the deposited layer (Goole and Amighi, 2016). FDM produces good X-Y axis resolution, however, the Z axis printing may impart some artifacts and it also relates to the thickness of the printed object, which may not necessarily be uniform (Lamichhane et al., 2019). Thus, a final step involving smoothen of the surface of the printed object may be necessary.

The versatility in FDM technique means that it has the potential to be used in achieving accurate dosing with relevance to PM. Moreover, the release profiles of incorporated APIs from 3DP dosage forms by FDM can be adjusted to suit a particular purpose, such as slow, delayed or pulsed release profile. Furthermore, FDM can be used to produce solid dosage forms that have rounded edges and corners, which improves the aesthetic appeal of the printed dosage form and consequently improves patient compliance (Schiele et al., 2013; Tan et al., 2018). Another advantage of FDM is the conversion of drug from crystalline to amorphous forms due to elevated temperatures required to generate the polymeric matrix (Solanki et al., 2018), the consequence of improved solubility.

However, the application of elevated temperatures (150–230°C) ascribable to FDM (Kollamaram et al., 2018) may not be applicable to thermolabile APIs and thermodegradable polymers, thus limiting the scope of its application (Lamichhane et al., 2019). However, Okwuosa et al. (2016) have successfully used polyvinyl pyrrolidone (PVP) to print theophylline and dipyridamole tablets at 110°C, lower than the recommended 230°C required for the 3DP. Kollamaram et al. (2018) have also used Kollidon^®^ VA64 (PVP-vinyl acetate copolymer) and Kollidon^®^ 12 PF (PVP) to print ramipril tablets at a lower temperature of 90°C. These studies indicate that the high temperature requirement for use of FDM in 3DP, which is a major constraint, may be overcome through careful selection of excipients and optimization such that, 3DP of dosage forms containing drugs with relatively low melting temperatures can be printed whist avoiding drug degradation.

Proper temperature setting is key for successful 3DP by FDM. Indeed, two major challenges faced in FDM are possible degradation of API due to elevated temperatures at which extrusion and printing are conducted and the availability of the right filament and excipients that would respond appropriately to set parameters (Lamichhane et al., 2019). The temperature setting during 3DP by FDM is mostly dependent on the polymer used, whereby thermoplastic polymers are recommended because of their relatively lower melting points. Moreover, the molten polymer should have a viscosity that is sufficiently high to permit object printing but low enough for allow extrusion. Polymers used in FDM include polyvinyl pyrrolidone (PVP) (Okwuosa et al., 2016), polylactic acid (PLA), polycaprolactone, polyvinyl alcohol (PVA), and polyethylene oxide (PEO) (Goyanes et al., 2016a; Melocchi et al., 2016; Ehtezazi et al., 2018).

Some water-soluble polymers used in FDM do not have appropriate printing properties. Consequently, another challenge in FDM is selection of suitable polymers for immediate-release products. This is because the API must be in its amorphous state to ensure better aqueous solubility. The printability of a filament depends on the filament diameter, rheological characteristics of the molten polymer, particularly at the printing temperature, and the thermal and mechanical properties of the polymer (Korte and Quodbach, 2018; Novák et al., 2018). Additionally, plasticizers, such as sorbitol, glycerol, polyethylene glycols, and triethyl citrate, may be needed if a filament is not printable. However, some APIs may serve as plasticizers, as has been observed for theophylline (Pietrzak et al., 2015; Melocchi et al., 2016; Palekar et al., 2019; Pereira et al., 2019). Plasticizers lower glass-transition temperature to improve the mechanical properties of polymers. Consequently, the temperature of extrusion/printing can be reduced (Vanin et al., 2005).

Additional parameters to be controlled during FDM are infill density, extruder speed, layer height, and the temperatures of the nozzle and build plate (Goyanes et al., 2014; Goyanes et al., 2015a; Goyanes et al., 2015c; Pietrzak et al., 2015; Skowyra et al., 2015). The infill density defines the amount of fill in an object and is related to the porosity of the printed object (Mohanty et al., 2015), which is typically in the range of 0–100, with 0 and 100% representing a completely hollow object and a completely solid object, respectively.

There are different FDM printers available with a wide range of versatility that can print objects to high precision, which is beneficial for producing specific dosage forms containing accurate drug doses for individual patients. Moreover, some FDM 3D printers have multiple nozzles for printing objects with complex geometries to achieve particular objectives (Lamichhane et al., 2019).

The feasibility of using FDM in the 3DP of pharmaceutical products has been shown in several studies. For example, fluorescein-loaded PVA filaments were used to fabricate a controlled release formulation where drug release occurred by erosion and was largely dependent on the infill percentage of the drug. Complete drug release took 20 h for a 90% infill tablet (Goyanes et al., 2014). Aminosalicylates (4-aminosalicylic acid [ASA] and 5-ASA) (Goyanes et al., 2015a) and prednisolone (Skowyra et al., 2015) tablets, as well as paracetamol, caffeine (Goyanes et al., 2016b), and budesonide (Goyanes et al., 2015b) caplets with controlled drug release characteristics have also been prepared by FDM 3DP using PVA as the polymer.

In the study by Skowyra et al. (2015), six types of ellipse-shaped tablets containing different doses of prednisolone were 3DP by FDM using prednisolone-loaded PVA filaments. The tablets exhibited extended drug release properties (up to 24 h). The drug contents of the tablets ranged from 2 to 10 mg. A good correlation between target and achieved dose was obtained (*R* ^2^ = 0.9904). The dose accuracy range was 88.7–107%. Thermal analysis and X-ray powder diffraction analyses indicated that the API existed mostly in its amorphous form within the tablets. Immediate-release caplet-shaped tablets of theophylline and dipyridamole have also been successfully prepared by FDM at a relatively low temperature (110°C) using the pharmaceutical grade polymer PVP, where drug release of more than 85% was achieved within 30 min (Okwuosa et al., 2016). This shows the flexibility of applying this technology to achieve desirable therapeutics.

Shell-core delayed release tablets containing theophylline have also been prepared by FDM 3DP. The tablets were enteric-coated; a dual-nozzle printer was used, in which one nozzle printed the API-containing core and another printed the shell (Okwuosa et al., 2017). In a study by Goyanes et al. (2015c), FDM was used to modify drug dissolution release profiles according to dosage form geometry. Five types of tablets with different shapes (cube, pyramid, cylinder, sphere, and donut) were printed using paracetamol as the model drug and evaluated. The investigators found that the release rate of the API was dependent on the surface area/volume ratio of the tablets. Specifically, 90% drug release was achieved in 2 h for the pyramidal tablets, which had the highest surface area/volume ratio, and at 12 h for the spherical tablets, which had the lowest surface area/volume ratio (Goyanes et al., 2015c). These findings show that tablets with complex geometries can be successfully fabricated using FDM 3DP, and that the dissolution profiles of APIs can be controlled based on tablet shape. This further points to the relevance of 3DP in formulating PM of solid dosage forms.

A unique dosage form for controlled release of hydrochlorothiazide was designed as a hollow cylinder with three compartments by Gioumouxouzis et al. (2017). The upper and lower layers of the cylinder were 3D printed by FDM with insoluble PLA filament, whereas the inner soluble drug-loaded compartment was printed with PVA (Gioumouxouzis et al., 2017). Similarly, hollow two-compartment cylinders with caps as a delivery device for two anti-tuberculosis drugs (rifampicin and isoniazid) have been designed and printed using PLA filaments and filled manually with the APIs. Physical separation of APIs and extended drug release were achieved (Genina et al., 2017).

Furthermore, liquid capsules have been formulated using a dual FDM 3D printer with a syringe-based liquid dispenser (Okwuosa et al., 2018). The investigators were the first to report of a fully automated process for 3DP of liquid capsules for immediate and extended release of dipyridamole and theophylline. Polymethacrylate was used to fabricate the shells. API dose and release profile were successfully controlled by manipulating shell thickness and the dispensed volume of API solution via the software used (Okwuosa et al., 2018). This further shows that FDM 3DP is a versatile technology that can be adapted to liquid-filled solid dosage forms. Consequently, in the clinical setting, small-volume liquid capsules can be easily prepared with individualized doses and release profiles in response to the needs of specific patients (Okwuosa et al., 2018).

Aside tablets and hollow cylinders, films have been fabricated by FDM 3DP. For instance, Jamróz et al. (2017) have printed orodispersible films containing aripiprazole as the API. Ehtezazi et al. (2018) have also successfully produced single-layered fast-dissolving oral films (FDFs) and multilayered FDFs using ibuprofen and paracetamol as the model drugs by the same technique. Filaments were prepared with PEO at 60°C for either drug, or with PVA for paracetamol at 130°C. The films were printed at 165°C (PEO) or 190°C (PVA) with promising disintegration times and drug contents (Ehtezazi et al., 2018).

From the foregoing, it is clear that FDM-based 3DP is a very promising technique that can be used to manufacture different types of solid dosage forms with varying and desirable characteristics. Drug release can also be modified to follow predetermined and specific profiles as indicated above. FDM can therefore be used to provide patient-tailored medicines to achieve the provisions of PM (*Personalized Medicine*). Importantly, the use of elaborate steps as mandated in the manufacture of conventional dosage form is not a requirement in FDM-based 3DP, therefore it is applicable in hospital dispensaries or pharmacies.

It has been demonstrated that immediate- and modified-release formulations can be successfully produced via FDM 3DP using filaments prepared with various polymers (Melocchi et al., 2016). However, the initial step of the process, which is filament production, is very crucial to ensure that target drug dissolution and release profiles are achieved. Melocchi et al. (2016) have successfully designed and produced various filaments using insoluble (ethylcellulose, Eudragit^®^ RL), promptly soluble (PEO, Kollicoat^®^ IR), enteric soluble (Eudragit^®^ L, hydroxypropyl methylcellulose acetate succinate), and swellable/erodible (hydrophilic cellulose derivatives, polyvinyl alcohol, Soluplus^®^) polymers that can be subsequently loaded with APIs for 3DP of dosage forms by FDM (Melocchi et al., 2016). In this study by Melocchi et al. (2016), the model APIs used were paracetamol and furosemide. Spools of such ready-made filaments, as prepared and evaluated by Melocchi et al. (2016), are typically used in the form of rollers.

#### Hot Melt Extrusion Fused With Fused Deposition Modelling in a Continuous Process

The polymers used in FDM 3D printers usually have to be in the form of filaments however, most filaments that currently available on the market are not suitable for pharmaceutical applications, which is a major obstacle for FDM 3DP of pharmaceutical products (Smith et al., 2018). Filaments used in FDM 3D printers must be of good quality to produce quality 3D printed products. Importantly, they should be able to withstand the mechanical and thermal stresses in the FDM print head to achieve a good printing quality (Dizon et al., 2018). Good quality thermoplastic filaments for FDM 3DP can be produced via HME. Specifically, the twin-screw extruder used in HME can be used to produce filaments of almost all types of polymers (Sadia et al., 2016). Moreover, due to the robustness of HME in producing different polymeric filaments for FDM 3DP, there is ongoing research on coupling HME with FDM 3DP for the manufacture of pharmaceutical products in a single continuous process. This can help to achieve a more effective and efficient manufacturing process, as the two technologies can streamline the complex processes of conventional manufacturing methods for pharmaceutical products. Combining these two processes into one also makes it possible to create different dosage forms for immediate use, which can be promising particularly in the clinical setting for PM. Moreover, HME fused with FDM can potentially make dosage form fabrication more cost-effective (Tan et al., 2018). Several dosage forms have been successfully produced via the coupling of HME with FDM (Zhang et al., 2017; Giri et al., 2020; Vo et al., 2020).

#### Inkjet Three-Dimensional Printing

Inkjet printing is a type of additive manufacturing that involves digitally-controlled formation and placement of small liquid drops onto a substrate using a pattern-generating device, followed by solidification. Typically, a combination of a powder and a binder “ink” is used to construct solid macroscopic structures in a layer-by-layer process. The process provides significant advantages for producing oral solid dosage forms and reducing the number of manufacturing steps as observed in conventional dosage form manufacture. Importantly, it can be used to fabricate unique pharmaceutical products for an individual patient. The two types of inkjet printing are continuous inkjet printing and drop-on-demand inkjet printing (Daly et al., 2015). Either process requires a printer head (thermal or piezoelectric) as part of the equipment setup and the need to control drop formation velocity and fluid viscosity for successful printing (Ihalainen, 2015).

3D inkjet printing enables precise deposition of a formulation on a substrate whilst offering the potential for significant scale up (Clark et al., 2017). One advantage of the inkjet technique over other printing techniques is that it facilitates a spatial resolution of up to 50 μm and co-deposition of multiple inks (Ibrahim et al., 2006; Hart et al., 2016; He et al., 2016). In 3D inkjet printing of pharmaceuticals, very small volumes (typically 10–500 pl) of the formulation ink can be deposited on the substrate (Daly et al., 2015). This has the advantage of spatial localization of materials and accurate delivery of the ink (Kyobula et al., 2017), which make the technique very useful for printing drug delivery devices containing accurate drug doses. Moreover, when tuning drug release based on the geometry of the printed object, there is no need to alter the formulation composition, manufacturing process, or equipment, which could significantly reduce production cost and time. Although dosage forms with simple geometries can easily be produced using traditional manufacturing equipment such as a tablet press, 3D inkjet printing offers great geometry flexibility whilst keeping low marginal costs when design changes are implemented (Gbureck et al., 2007; Berman, 2012; Beyer, 2014; Gebler et al., 2014). Moreover, 3D inkjet printing can also be used to modify initial drug distribution within solid dosage forms such as tablets to control drug release rates (Kyobula et al., 2017).

Vuddanda et al. (2018) and Thabet et al. (2018) have used inkjet 3DP technique to print warfarin and hydrochlorothiazide, respectively, onto substrate films in order to obtain specific doses of the drugs. Complex dosage regimes are usually required for drugs with narrow therapeutic indices and large inter-individual variability, such as warfarin. Inkjet 3DP technique can therefore be potentially used for PM and simplifying the administration of such drugs.

Kyobula et al. (2017) were the first to produce a viable and tuneable pharmaceutical dosage form by the 3D inkjet printing technique. In the study, a solvent-free 3D hot-melt inkjet printing method was used to fabricate solid dosage forms with variable complex geometries at high spatial resolution to achieve variable and predictable drug release profiles. Additionally, an analytical model based on Fickian diffusion was developed for the prediction of drug release to facilitate the selection of geometries with suitable configurations. This is very important because such a model may be useful in defining the geometry required to produce a clinically specific drug release profile. Tablets with a honeycomb geometry containing fenofibrate as the model API were successfully printed by the investigators. The method may be similarly used for the production of customized drug delivery devices (Kyobula et al., 2017).

Rowe et al. (2000) have also used inkjet 3DP to fabricate four types of complex devices for oral drug delivery: immediate-extended release tablets composed of two drug-containing sections, breakaway tablets, enteric dual pulsatory tablets, and dual pulsatory tablets. In a recent study by Clark et al. (2020), the viability of 3D inkjet printing combined with UV curing was used to produce solid dosage forms containing the poorly water-soluble drug carvedilol. Characterization of the printed tablets showed that the drug was in its amorphous state. Dosage forms with different geometries (ring, mesh, cylinder, thin film) were successfully printed and the surface area/volume ratio of each was estimated. Over 80% carvedilol release was observed for all printed tablet geometries within 10 h, with the fastest drug release from the thin films, followed by the ring and mesh geometries, and slowest in the cylindrical forms (Clark et al., 2020).

Furthermore, solvent inkjet printing has been used to print tablets containing PVP as the model excipient and thiamine hydrochloride as the model drug. A water-based ink formulation was developed that exhibited reliable and effective jetting properties. The tablets were printed on polyethylene terephthalate films. The drug content in the tablets was found to be commensurate with that in the ink formulation. The printed tablets displayed rapid drug release. The strategy developed Cader et al. (2019) for tablet manufacturing from a suitable ink provides a framework for the formulation for any drug that is soluble in water (Cader et al., 2019). Clark et al. (2017) have also used piezo-activated inkjetting to 3D print tablets containing 0.41 mg of ropinirole hydrochloride.

These studies indicate the possibility of translating scalable, high precision, and bespoke inkjet-based 3DP to the pharmaceutical sector. Crucially, the technique can be used to produce dosage forms for personalized dosing to meet the specific patient needs. However, there are some challenges associated with the technique. Generally, the density, viscosity, and surface tension of the ink must be optimum within narrow ranges for effective printing. However, these characteristics largely depend on the carrier fluid since it forms a significant proportion of the ink. One challenge with inkjet printing is the correct choice of carrier fluid (Daly et al., 2015). Presently, common carrier fluids used in inks are water, ethanol, dimethyl sulfoxide, and acetone. These fluids are normally used as vehicles or solvents; however, they can also be used to control evaporation of the ink to prevent nozzle blockage and ensure that the desired product morphology is achieved (Daly et al., 2015). In inkjet printing of pharmaceuticals, inks must have viscosities in the range of 2–20 mPa s, although a viscosity of approximately 10 mPa s is required for most printers to prevent nozzle blockage and ensure effective ink deposition (Martin and Hutchings, 2013; Daly et al., 2015).

Another concern in inkjet printing is API adsorption onto the internal surfaces of large ink reservoirs containing slow-moving fluid (Daly et al., 2015). Leaching of materials from the printing equipment into the ink formulation can also occur in this technique, which may affect the stability or viscosity of the formulation (McDonald et al., 2008). Additionally, ink formulations that are nanosuspensions or nanoemulsions may exhibit flocculation, settling, creaming, aggregation, and Ostwald ripening (Grau et al., 2000; Daly et al., 2015), which can result in nozzle blockage. Products printed from such formulations may not be homogeneous with respect to API concentration (Daly et al., 2015).

#### Laser-Based Printing

##### Stereolithography

SLA is a non-thermal, laser-based printing technology developed in 1986 (Melchels et al., 2010). It involves controlled solidification of a liquid resin or polymer solution by photopolymerization using a controlled UV-laser source (Karakurt et al., 2020). The laser is focused on a specific area to densify, and solidification is repeated layer-by-layer to obtain the desired object (Wang et al., 2016; Fina et al., 2017). Photopolymerization in SLA can be achieved via cationic polymerization (mostly with vinyl ether, epoxides, or lactones) or radical polymerization (mostly with acrylate derivatives) (Andrzejewska, 2001). Typically, polymerization occurs in a chain reaction once monomers are activated. The process is normally started with a photoinitiator, which must hardly absorb in the wavelength range of the laser in order to provide excited states with a very short life time to avoid deactivation by oxygen (Dietliker, 1991; Jennotte et al., 2020).

One key parameter that must be controlled in SLA printing is the thickness of the cured layers, which is mainly influenced by the light energy to which the resin is exposed (Melchels et al., 2010). Resins used in the technique must be carefully selected, as they should form liquids that can be solidified upon exposure to UV light. Low-molecular-weight polyacrylate macromers appear suitable for the fabrication of drug delivery systems via SLA (Goole and Amighi, 2016).

Advantages of this 3DP technology include short print times, production of objects with high surface finish and resolution at room temperature, production of elaborate shapes, and suitability for thermolabile drugs. However, limitations such as toxicity of the photopolymerizing material require further studies (Fina et al., 2017; Kyobula et al., 2017; Karakurt et al., 2020).

Wang et al. (2016) have used SLA to fabricate tablets with extended-release characteristics using 4-ASA and acetaminophen as the model drugs. The tablets were printed in a torus shape, which is complex to achieve via conventional tablet manufacturing, to indicate how the technique can be used to manufacture complex and customized dosage forms. The monomer and photoinitiator used were polyethylene glycol diacrylate (PEGDA) and diphenyl(2,4,6-trimethylbenzoyl)phosphine oxide (TPO), respectively. The torus shape was found to help increase the aqueous solubility of the formulations due to increased surface area. Additionally, the APIs did not degrade but had increased solubility because they were in their amorphous forms (Wang et al., 2016). Martinez et al. (2018) have also used SLA to manufacture paracetamol-loaded printlets with different shapes (cube, disc, pyramid, sphere, and torus). PEGDA and TPO were used as the photopolymerizable resin and photoinitiator, respectively. The investigators found that printlets with constant surface area to volume ratio showed the same drug release rate. This clearly indicates that dissolution behavior can be adjusted by varying the surface area to volume ratio of printlets to obtain personalized dosage forms.

Similarly, in another study (Robles-Martinez et al., 2019), PEGDA and TPO were used to fabricate cylindrical and ring-shaped multi-layer constructs (polypills) by SLA 3DP. Remarkably, six different drugs (aspirin, caffeine, chloramphenicol, naproxen, paracetamol, and prednisolone) were used as model drugs and co-loaded in the constructs. The order of the drugs was maintained in all the printed structures. APIs with higher water solubility were printed as the inner layers, whereas those with the lowest water solubility were printed as the outer layers. It was found that some of the drugs diffused into other layers depending on their amorphous or crystalline phase. However, the findings clearly show that SLA can be used to print polypills for personalized dosing to improve polypharmacy (Robles-Martinez et al., 2019).

Recently, Xu et al. (2020) fabricated multi-layer dosage forms (polyprintlets) loaded with four antihypertensive drugs (amlodipine, atenolol, hydrochlorothiazide, and irbesartan) by SLA. The polyprintlets were successfully fabricated. However, there was an unexpected chemical reaction between the photopolymer used (PEGDA) and amlodipine, which shows that similar for conventional dosage forms, photocurable resins as excipients must be carefully selected for SLA 3DP. Karakurt et al. (2020) have also fabricated hydrogels via the SLA 3DP technique using ascorbic acid as the model drug and poly(ethylene glycol) dimethacrylate as the polymer. Riboflavin was used as a non-toxic photoinitiator. Tablets with different geometries (surface area to volume ratios ranging from 0.6 to 1.83; coaxial annulus, 4-circle pattern, and honeycomb pattern) were successfully fabricated. The formulations with the honeycomb and coaxial annulus geometries showed higher *in vitro* drug release rates of about 80% within 1 h (Karakurt et al., 2020).

##### Selective Laser Sintering

SLS is another laser technique for 3DP, in which a laser beam is used to sinter powder particles together. It is a high resolution and single-step printing technology that can be used to fabricate very detailed structures with immediate or modified drug release characteristics via a unique binding thermal process (Shirazi et al., 2015; Fina et al., 2018). During printing, the laser is directed to draw a specific pattern on the surface of a powder bed. After the first layer is completed printed, a new layer of powder is distributed on top of the previous one followed by sintering. Consequently, objects are fabricated layer-by-layer process and then recovered from underneath the powder bed (Fina et al., 2017). One advantage of the technique is that the unsintered powder could be reused to minimize wastage. Additionally, SLS is purported to be more cost-effective than FDM and SLA, and can be used to construct structures that may be difficult to fabricate using other techniques (Hopkinson and Dicknes, 2003; Fina et al., 2018).

Fina et al. (2018) have used SLS to fabricate 3D gyroid lattice constructs, which are porous solids with a broad range of micro-structures and length scales (Khaderi et al., 2014), as well as cylindrical and bi-layer structures having customizable drug release profiles. Paracetamol was used as the model drug in the study, whereas PEO, Eudragit (L100-55 and RL), and ethyl cellulose were the polymers investigated (Fina et al., 2018). In an earlier study by Fina et al. (2017), SLS was used to construct a solid dispersion of paracetamol using Kollicoat^®^IR and Eudragit^®^ L100-55. No API degradation was observed, and increasing API amount resulted in structures that were less porous and released the loaded API more slowly. The findings show that SLS could be a useful and practical 3DP technology for producing dosage forms (Fina et al., 2017).

Allahham et al. (2020) have fabricated cylindrical ondansetron-loaded orodispersible printlets via SLS 3DP and evaluated them against a commercial ondansetron orally disintegrating tablet containing the same API dose. The drug was complexed with cyclodextrin. Mannitol and Kollidon^®^ VA-64 were used as filler and polymer, respectively. The printlets disintegrated in approximately 15 s and achieved >90% drug release within 5 min, with the commercial product producing similar data (Allahham et al., 2020), which is a clear indication that drug performance is not affected by 3DP. Amorphous solid dispersions and printed dosage forms containing ritonavir have also been prepared using a single-step SLS 3DP method by Davis et al. (2020). Characterization of the ASDs revealed that the crystalline form of the API had been fully converted to its amorphous form. Optimum process and formulation parameters such as powder flow, surface and chamber temperatures, laser speed, and hatch spacing were found to be crucial for successful formation of the ASDs. Interestingly, API solubility in the printed tablets was found to have increased 21-fold (Davis et al., 2020). In a more recent study, Hamed et al. (2021) fabricated circular lopinavir printlets via SLS 3DP, achieving a high API content of 95.2–100.9. The tablets disintegrated within 2 min with >90% of the drug released in <30 min. It was found that printlet porosity increased as lopinavir amount was increased. The actual quantities of the drug in its amorphous and crystalline forms in the printed structures were found to be close to the predicted values (Hamed et al., 2021). These studies evidently show that the SLS 3DP technique is a feasible and potentially valuable technology for fabricating patient-specific dosage forms for individualized therapy. Importantly, API degradation was insignificant or not observed in these studies, which show that careful control and finetuning of process parameters such as surface temperature and laser speed may prevent API degradation.

## Quality Assurance and Regulatory Controls in 3D Printed Tablets

### Quality Assurance

Quality assurance on 3D printed tablets, like other dosage forms, must ideally commence on the raw materials used and should involve all steps through the manufacturing process to final testing of the finished product. Of the various techniques explored within the realm of 3DP of tablets, FDM and possibly HME Fused with FDM emerges with the most consequential implication to the concept of PM (*Personalized Medicine*). Yet, there is currently no commercially available 3D tablet printer dedicated to personalized medicines on the market (Araújo et al., 2019). In either of the two techniques, the filament is the key input material, which can be impregnated with drug during mixing, prior to hot melt extrusion and packaging as spool units. Quality assurance on the performance of the filament in response to anticipated stresses during printing should be ascertained. These may include rheological responses of the filament to variable temperature and compression (extrusion) forces. Size changes in filament during printing can significantly impact on its flow characteristics through the nozzle. In inkjet deposition, material is jetted onto a platform and rapidly solidifies into the desired structure. Jetting material may comprise of suspensions, waxes, and solutions containing the API (Norman et al., 2017). As in FDM, the rheological responses of the jetting material to anticipated stresses should be qualitatively assured and ascertained. The solidification time after jetting and the physical properties of the printed tablet should also be assured.

In all cases, the *in vitro* release of the API from the printed tablet gives a measure of its performance *in vivo*. Quality assurance on release of API may be conducted similarly to conventional tablets, using the United States Pharmacopoeia dissolution apparatus. The amount of API released over a specified time frame may be used as a criterion to assure quality. To meet the provisions of personalization of the printed tablets, the release of the API from the tablets should be assured as regards to the desired response in the patient in question, which may include immediate release, delayed or slow release. In addition, the hardness of the printed tablets and friability should be conducted to give assurance on the response of the tablets to handling and usage.

### Regulatory Controls

There is growing interest by stakeholders in the application of 3DP in healthcare, and with this growing interest comes the need for regulatory controls governing the production and use of printed products. It is inconceivable that PM would emerge as a key beneficiary to 3DP technology because of the huge potential it holds to yielding optimal therapeutics and filling some of the gaps associated with current medical treatment modalities. Regulatory authorities such as the FDA are cognizant of this huge potential but a key distinction is to be drawn on current conventional production of medicines and the evolution of personalized medicines, which aptly, will apply to the compounding of medicines in the pharmacy settings (Sun, 2013; Sparrow, 2014). The application of 3DP in PM also presents ambiguity on the legal framework upon which authorities must apply controls to the production of medicines. Furthermore, issues related to intellectual property and tort liability would need to be contended with (Alhnan et al., 2016). There is also the question of which regulatory pathways researchers/inventors must follow in the production of PM. Notwithstanding, there appears to be traction in the realization of and support by regulatory authorities for *p*PM. Currently, several 3D printed medical devices have gained clearance by the FDA (Sun, 2013). Furthermore, “fast-track” routes to obtaining clearance for 3D printed devices such as the 510(k), established the notion that 3D printed products are significantly equivalent to legally marketed devices (United States Food and Drug Administration, 2020). However, there is yet to be a similar “fast-track” route for 3D printed dosage forms, but as mentioned earlier, it is only a matter of time that similar approval pathways emerge for printed dosage forms. Spritam^®^ (containing levetiracetam and manufactured by Aprecia Pharmaceuticals Company) is the first and only 3D printed dosage form currently on the market (Dodziuk, 2016). We envisage that more 3D printed dosage forms would emerge on the market in the near future and this would be paralleled with the perfection in 3D printing technologies for PM.

## Practicality of Three-Dimensional Printing in Personalized Medicine

### Pharmacological Basis of Diagnosis

Polymorphic differences in DNA and resultant genetic expressions have paved the way for a wide range of clinical presentations of the same disease in different individuals. Several failed clinical trials are based on lack of information on the genetic expression of participants. In the same vein, patients would be better served if clinicians are privy to genetic data during routine diagnosis and prescribing. Genetic responses to medication is complex and often not determined by a single gene but a combination (Mancinelli et al., 2000). In the context of the practicality of 3DP for PM, some progress has been made by Prescription Benefit Management (PBM) companies to expand their genetic testing as part of the prescription filling process. Furthermore, genetic testing is now an option as part of treatment with some commercially prescribed APIs such as warfarin (Coumdin by Bristol-Myers Squibb) and tamoxifen (Nolvadex, AstraZeneca). Obviously, the decision to take a genetic test during prescription is not mandatory and depends heavily on insurance provisions. Privacy restrictions also limits the extended use of genetic testing during prescribing (Haile, 2008). Finally, stakeholders are aware of the interconnectivities of each modality in the realization of 3DP in PM and steps toward this realization have begun, albeit slowly.

### Cost of Printing for Personalized Medicine

As the 3DP technology is still emerging, printing of tablets is less cost effective compared to conventional manufacturing. For example, the cost for the development of Spritam^®^ is higher than commercially available brands (Lamichhane et al., 2019). In the context of PM, associated costs on genetic testing may further add to the overall cost for dispensed 3DP tablets and thus, be prohibitive to patients. The availability of appropriate materials for use in printing of dosage forms with desirable drug release characteristics may also pose a challenge. The rate of production of 3D printed tablets is a key feature with consequential implications to PM (Pietrzak et al., 2015). The application of 3DP technology in PM within hospital dispensaries and community pharmacies is still conceptual as regulatory and financial barriers slowly begin to be surmounted. However, the practical framework is very feasible, where individualized clinical data can be sent electronically to a 3D printer for printing.

### Quality of Printed Tablets in Personalized Medicine

Quality control on conventional dosage forms follow a set of well-defined testing protocols during production and on finished products aimed at assuring the desired quality to the end-user. In 3DP of tablets within the hospital dispensary or community pharmacy settings, performing quality control tests during and on printed tablets prior to dispensing may be prohibitive as regards waiting time by patients. In addition, there are quality assurance issues akin to specific 3DP technologies. For example, 3DP by extrusion may result in shrinking or deformation of the printed tablet during drying. Clogging of printer nozzle (*Quality Assurance and Regulatory Controls in 3D Printed Tablets*) has been a recurring constraint in most 3D printers. The stability of APIs in 3D printed tablets made by FDM needs assurance (Goyanes et al., 2015a). Reports on degradation of prednisolone (Skowyra et al., 2015), 4-ASA, and 5ASA (Goyanes et al., 2015a), among other drugs, due to exposure to elevated temperature during FDM bears testament to the importance of quality assurance on printed tablets. The emergence of “rapid” testing technologies based on near infrared and Raman spectroscopy provide fairly accurate information on the characteristics of APIs and may thus hold the key to some quality assurance on 3DP tablets within the PM framework (Trenfield et al., 2018). Tests aimed at gauging the release profile of the API from printed tablets are also warranted. Several release profiles are achievable from 3DP such as dual-pulsed (Thakral et al., 2013) and sustained release (Rowe et al., 2002), among others. Whilst these release profiles have desirable clinical implications, the practicality of employing quality control on printed tablets within the PM settings has yet to be accomplished.

## Conclusion/Future Direction

The fit-for-all treatment modality associated with conventional therapeutics does not address the full scope of clinical presentations from patients. In fact, it does present adverse effects in some individuals whilst at the same time eliciting sub-therapeutic levels in others. Such differences in responses to therapeutics has prompted the emergence of the concept of PM, which in addition, opens the door to the utilization of previously approved drugs that have limited market value due to toxicity concerns. Such drugs might hold key to manifestation of resistance through application of appropriate dose adjustments guided by genetic predispositions in patients. However, PM faces several constraints in the march towards full realization. The concept of 3D printing of dosage forms at dispensaries and pharmacies offers a window into the possibility for clinicians and pharmacists to calibrate therapeutic doses required by patients based on their pharmacogenetic profiles. The idea of applying 3D printing to PM is slowly evolving because of interconnectivity with legal provisions surrounding privacy of personal information, regulatory requirements, cost, and practicalities. For a meaningful implementation of this concept to materialize in the foreseeable future, researchers, practitioners and policy-makers must work in concert in addressing hurdles. The FDA is cognizant of the interplay between stakeholders and is supportive a collaborative effort. A complete replacement of current conventional therapeutics by 3DP in PM will not be feasible, nor is it necessary. It is possible for both modalities to apply in therapeutics.
